# FDG-PET metabolic response predicts outcomes in anal cancer managed with chemoradiotherapy

**DOI:** 10.1038/bjc.2011.274

**Published:** 2011-07-26

**Authors:** F L Day, E Link, S Ngan, T Leong, K Moodie, C Lynch, M Michael, E de Winton, A Hogg, R J Hicks, A Heriot

**Affiliations:** 1 Department of Haematology and Medical Oncology, Peter MacCallum Cancer Centre, Locked Bag 1, A’Beckett Street, Melbourne, 8006, Victoria, Australia; 2 Centre for Biostatistics and Clinical Trials, Peter MacCallum Cancer Centre, Locked Bag 1, A’Beckett Street, Melbourne, 8006, Victoria, Australia; 3 Department of Radiation Oncology, Peter MacCallum Cancer Centre, Locked Bag 1, A’Beckett Street, Melbourne, 8006, Victoria, Australia; 4 Centre for Molecular Imaging, Peter MacCallum Cancer Centre, Locked Bag 1, A’Beckett Street, Melbourne, 8006, Victoria, Australia; 5 Department of Surgical Oncology, Peter MacCallum Cancer Centre, Locked Bag 1, A’Beckett Street, Melbourne, 8006, Victoria, Australia

**Keywords:** anal cancer, chemoradiotherapy, metabolic response, FDG-PET

## Abstract

**Background:**

The aim was to investigate the correlation between ^18^F-fluorodeoxyglucose positron emission tomography (FDG-PET) metabolic response to chemoradiotherapy and clinical outcomes in squamous cell carcinoma (SCC) of the anus.

**Methods:**

A total of 48 patients with biopsy-proven anal SCC underwent FDG-PET scans at baseline and post chemoradiotherapy (54 Gy, concurrent 5-FU/mitomycin). Kaplan–Meier analysis was used to determine survival outcomes according to FDG-PET metabolic response.

**Results:**

In all, 79% patients (*n*=38) had a complete metabolic response (CMR) at all sites of disease, 15% (*n*=7) had a CMR in regional nodes but only partial response in the primary tumour (overall partial metabolic response (PMR)) and 6% (*n*=3) had progressive distant disease despite CMR locoregionally (overall no response (NR)). The 2-year progression-free survival (PFS) was 95% for patients with a CMR, 71% for PMR and 0% for NR (*P*<0.0001). The 5-year overall survival (OS) was 88% in CMR, 69% in PMR and 0% in NR (*P*<0.0001). Cox proportional hazards regression analyses for PFS and OS found significant associations for incomplete (PMR+NR) *vs* complete FDG-PET response to treatment only, (HR 4.1 (95% CI: 1.5–11.5, *P*=0.013) and 6.7 (95% CI: 2.1–21.6, *P*=0.002), respectively).

**Conclusion:**

FDG-PET metabolic response to chemoradiotherapy in anal cancer is significantly associated with PFS and OS, and in this cohort incomplete FDG-PET response was a stronger predictor than T or N stage.

Squamous cell carcinoma (SCC) of the anus is an uncommon malignancy, but increasing in incidence (Johnson *et al*, 2004; Robinson *et al*, 2009). Aetiologic factors include infection by human papilloma virus (Frisch *et al*, 1997) and/or human immunodeficiency virus (Palefsky *et al*, 1998) and smoking (Holly *et al*, 1989). Historically, anal cancer was managed surgically with abdominoperineal resection (APR), but since the landmark publication by Nigro *et al*, 1974, chemoradiotherapy has been the mainstay of treatment. This management approach allows preservation of the anal sphincter, avoidance of permanent colostomy and prophylactic radiotherapy to the regional nodal basins. The all-stage 5-year overall survival (OS) with current therapy is 58% (Bilimoria *et al*, 2009).

Staging of anal cancer by clinical assessment (primary and inguinal nodes) was later supplemented by the use of computed tomography (CT) (Scherrer *et al*, 1990) and, in some cases, additional anatomic imaging of the pelvis with magnetic resonance imaging or ultrasound. Multiple recent studies have now demonstrated the utility of ^18^F-fluorodeoxyglucose positron emission tomography (FDG-PET) in detecting inguinal or pelvic node involvement not evident clinically or on CT (Trautmann and Zuger, 2005; Cotter *et al*, 2006; Nguyen *et al*, 2008; Iagaru *et al*, 2009; de Winton *et al*, 2009), with subsequent impact on the planning of definitive radiotherapy fields (Nguyen *et al*, 2008; de Winton *et al*, 2009). The FDG-PET staging of anal cancer has become the standard of care in many centres.

Post-treatment FDG-PET assessment of tumour metabolic response is increasingly employed in treatment protocols for Hodgkin's and non-Hodgkin's lymphomas (Juweid *et al*, 2007), non-small cell lung cancer (MacManus *et al*, 2003), oesophageal (Duong *et al*, 2006) and head and neck cancers (Andrade *et al*, 2006). For some FDG-avid malignancies, FDG-PET has been demonstrated to be more accurate than CT alone in the evaluation of tumour response to therapy (MacManus *et al*, 2003; Andrade *et al*, 2006). Tumour metabolic response has also been demonstrated to provide robust prognostic information, particularly in lymphoma where both positive and negative predictive values for interim and post-treatment FDG-PET scans and disease progression are high (Mikhaeel *et al*, 2000; Kobe *et al*, 2008). Currently, assessment of response to chemoradiotherapy in anal carcinoma relies on serial clinical examination from 8 to 12 weeks post chemoradiotherapy (NCCN Clinical Practice Guidelines in Oncology, V.1.2010). However, clinical estimation of tumour response is subjective and may be confounded by radiotherapy-related skin toxicity (Borzomati *et al*, 2005) and residual nonmalignant masses (Sato *et al*, 2005). Histopathological interpretation of biopsies performed for nonresolving lesions or new in-field changes may also be difficult in the setting of post-radiotherapy change.

Our aim was to investigate the clinical significance of FDG-PET metabolic response to radical chemoradiotherapy in SCC of the anus. FDG-PET responses to treatment were evaluated and correlated with the end points of progression-free survival (PFS), OS and cause-specific survival. We also examined the prognostic power of post-therapy FDG-PET in comparison with the known anal cancer prognostic variables of baseline tumour T and N stage (Deniaud-Alexandre *et al*, 2003; Bilimoria *et al*, 2009; Myerson *et al*, 2009).

## Materials and methods

### Study population

Patients with a diagnosis of anal carcinoma were retrospectively identified from our institutions’ PET Centre electronic research database. Incorporation within the database signifies receipt of one or more PET scans, and provision of individual patient consent to data use for research purposes. Institutional ethics committee approval was granted for research applications of the database. Patient records were then manually reviewed to ensure histologic confirmation of anal carcinoma, planned radical chemoradiotherapy and performance of a baseline pretreatment FDG-PET scan. A total of 74 patients met these criteria between September 1997 and April 2006. The impact of baseline FDG-PET scan results on the staging and radiotherapy field planning of a proportion of these patients (61 patients, from September 1997 to November 2005) has been previously reported (de Winton *et al*, 2009). Patients within the cohort who additionally underwent post-chemoradiotherapy FDG-PET assessment of tumour response (*n*=48) were identified for this study and all included for analysis.

### FDG-PET imaging and interpretation

Both pre- and post-treatment FDG imaging was with dedicated PET in 20 patients, hybrid PET-CT in 20 patients and one scan of each type in 8 patients. PET scans were acquired on a GE QUEST 300-H (3D mode, sodium iodide detector) scanner (UGM Medical Systems, Inc., Philadelphia, PA, USA) and PET-CT scans on a GE DLS PET-CT (2D mode, Bismuth Germanate detector) scanner (Discovery LS; GE Healthcare, Waukesha, WI, USA). The PET images were acquired at least 1 h after intravenous injection of 80–120 MBq of ^18^F-FDG on the GE Quest scanner and 300–400 MBq on the GE DLS scanner. All patients fasted for 6 h before the PET study but were encouraged to drink water. Patients were catheterised and administered furosemide 30 min before imaging to minimise bladder activity, and also received bowel preparation. The blood glucose level of the patients was required to be <10 mmol l^–1^ before FDG administration.

Transmission and emission scans were obtained from the lower neck to the upper thighs. Emission data were processed using OSEM (ordered-subset expectation maximisation method) reconstruction. Attenuation correction with single transmission source (Benard *et al*, 1999) was performed on the GE Quest scanner and with CT data on the GE DLS scanner. Data sets were reported both with and without attenuation correction. Rotating count-rendered images were also reviewed to aid clarification of the relationship between the physiological radiotracer accumulation and tumour in the anus.

All PET and PET-CT studies were reported on the day of the scan by experienced PET specialists. Pretreatment PET images were available for unblinded review and comparison during post-treatment scan interpretation. For chest, abdominal and pelvic activity, abnormal focal uptake of ^18^F-FDG had to be greater than mediastinal uptake, and needed to correspond to an anatomical structure or abnormality identified on CT; for example, a lymph node of normal or abnormal size. Any ^18^F-FDG activity less than mediastinal blood pool activity was defined as abnormal only if there was a definite corresponding structural abnormality of <1 cm in size (because of the known partial volume effect of PET-CT caused by its limited resolution below 1 cm). Increased uptake in the radiotherapy field in a geographic distribution or not conforming to anatomical structures involved at baseline was assumed to represent post-radiotherapy changes as previously described (Kalff *et al*, 2009).

A complete metabolic response (CMR) to treatment was defined as a return of visually graded ^18^F-FDG uptake in all baseline lesions to a level equivalent to or lower than the radioactivity in normal tissues of the involved organ, as previously described (MacManus *et al*, 2003; Hicks, 2009). Partial metabolic response (PMR) was defined as an improvement in visually graded ^18^F-FDG uptake at baseline involved sites, but persistent residual abnormality suggesting malignancy, and additionally included equivocal scans where residual disease could not be excluded. No response (NR) to treatment was defined as no change or an increase in ^18^F-FDG uptake within a baseline lesion, consistent with tumour growth, or development of a new site of disease. The worst response at any site was used for categorisation.

### Chemoradiotherapy

All patients received definitive external beam radiotherapy with concurrent chemotherapy. Radiotherapy was delivered using 6–18 MV photons to a total dose of 54 Gy in 1.8 Gy daily fractions, five fractions per week using a three-phase technique. Phase 1 consisted of anterior–posterior parallel opposed fields, with the clinical target volume (CTV) covering the primary tumour, perirectal, iliac and inguinal lymph nodes to a total dose of 36 Gy. Phase 2 consisted of a three-field technique, with the CTV encompassing the primary tumour and anal canal, perirectal and iliac nodes to a total dose of 45 Gy. Phase 3 consisted of a reduced three-field technique that boosted the primary tumour and anal canal to a total dose of 54 Gy. Any involved inguinal or pelvic nodes were also boosted to 54 Gy. Patients with stage I disease did not receive prophylactic irradiation of the inguinal nodes. Information from the baseline PET scan was used to assist in radiotherapy treatment planning.

Concurrent chemotherapy was with mitomycin C 10 mg m^–2^ intravenously on day 1, and either 5-fluorouracil (5-FU) 1 g m^–2^ day^–1^ continuous intravenous infusion for 4 days during weeks 1 and 5 of radiotherapy or an alternative infusional 5-FU schedule of 300 mg m^–2^ day^–1^ for 4 days of every week of radiotherapy on a clinical trial. The median duration of chemoradiotherapy was 39 days.

### Post-therapy assessment and follow-up

Patients were evaluated clinically for tumour response at regular intervals post chemoradiotherapy as per usual practice. Post-treatment FDG-PET scans were performed at a median of 69 days after the final radiotherapy fraction (range 20–255 days). As early post-therapy FDG-PET scans may be anticipated to be less specific for persistent disease due to acute radiotherapy change, and late scans delay evaluation of response and are potentially influenced by pretest selection bias for patients with symptoms or signs suggesting possible relapse, the subgroup of patients undergoing PET scans within 40–140 days post treatment (*n*=34, 71% cohort) were the subject of a separate analysis (Table 2). Some patients underwent two post-therapy scans because of equivocal results for residual disease in the first scan. In this group, the second scan result was utilised for analysis but only if it occurred <140 days after treatment completion, and this was the case for 7 patients (15% overall).

Long-term follow-up for determination of cancer-specific outcomes was via patient attendance at hospital clinics or through the referring physician. Final patient follow-up occurred between February 2009 and January 2010 for 29 of the 35 patients not known to have died. Median patient follow-up among these 35 patients was 5.0 years (range 1.7–9.1 years). Disease progression was confirmed on biopsy for all patients with local recurrence and imaging ±biopsy in the case of distant metastasis.

### Statistical analysis

Fisher's exact tests and exact Cochran–Armitage tests for trend were used for the comparison of patients with and without post-treatment PET scans; *P*-values were two sided. The 95% confidence intervals were exact. Progression-free survival was measured from the date of final radiotherapy fraction until confirmed anal cancer progression or death from any cause. Overall survival and anal cancer-specific survival (CSS) were measured from the date of final radiotherapy fraction to date of death.

The PFS, OS and CSS were estimated using the Kaplan–Meier method. Univariate Cox proportional hazards regression modelling was used to determine the association of tested variables with PFS and OS. Analyses were performed using SAS (v9, SAS Institute, Cary, NC, USA).

## Results

Of the 74 patients who received staging FDG-PET scans, 48 (65%) also underwent post-treatment FDG-PET assessment of tumour response. The characteristics of this group in comparison with the entire cohort are shown in Table 1. To exclude selection bias among patients undergoing both pre- and post-therapy FDG-PET scans, we compared patient demographics, T stage, N stage and stage grouping with those of the entire cohort; only T stage was shown to be significantly different (*P*=0.02) because of the higher representation of T2 and T4 tumours in patients undergoing both studies.

## Pre- and post-chemoradiotherapy FDG-PET results

Baseline tumour staging is shown in Table 1. Pre-treatment FDG-PET results according to site were: 45 patients with FDG-avid primary lesion (94% 3 patients without an avid primary lesion due to pre-PET excisional biopsy); 15 with FDG-avid regional lymph node involvement (31%); and 2 with metastatic disease (4% 1 with para-aortic node involvement and 1 with low-volume supraclavicular and axillary lymphadenopathy. In addition, 15 patients had coincident nonanal carcinoma distant FDG-avid abnormalities; most commonly, inflammatory mediastinal lymphadenopathy consistent with granulomatous disease (6 patients, 13%) and thyroiditis (3 patients, 6%). However, for 3 of these 15 patients (6% of the cohort), the coincidental abnormality represented a synchronous malignancy: two primary lung cancers and one bladder cancer.

Metabolic responses to chemoradiotherapy are shown in Table 2. Overall, 94% of patients experienced a metabolic response to chemoradiotherapy (79% CMR, 15% PMR) in both PET scan ranges.

### FDG-PET metabolic response and survival outcomes

At the time of analysis, 11 patients had experienced disease progression: 5 with local recurrence only and 6 distant relapse. The patients categorised as metabolic nonresponders (*n*=3) all manifested progressive distant disease, two with para-aortic lymphadenopathy only and one with liver metastases. A total of 13 patients (27%) are known to have died, 7 from anal cancer and 6 because of other causes, although nil from the coincident malignancies at baseline.

**Figure 1:**
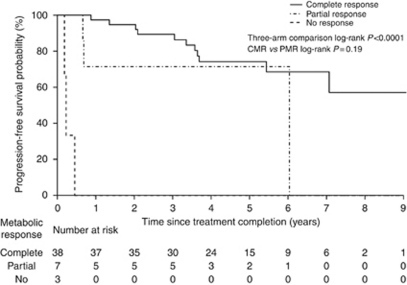
Progression-free survival in all patients according to FDG-PET metabolic response.

The PFS stratified for FDG-PET metabolic response is shown in Figure 1. At 2 years, estimated PFS for patients experiencing a CMR was 95% (95% CI: 88–100%), for PMR 71% (95% CI: 45–100%) and for NR 0% (95% CI: 0–71%). There was a significant difference between the three categories of PET response (*P*<0.0001), but not PFS according to the degree of metabolic response (CMR *vs* PMR; *P*=0.19). This analysis was repeated for the subgroup with post-therapy scans within 40–140 days with similar results; *P*<0.0001 for the three-arm comparison and no significant difference for CMR *vs* PMR (data not shown).

**Figure 2:**
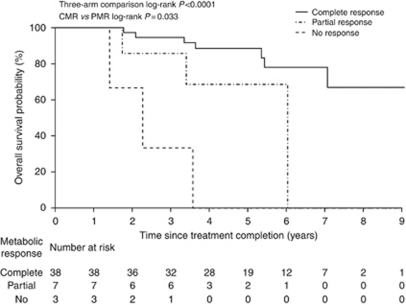
Overall survival in all patients according to FDG-PET metabolic response.

**Figure 3:**
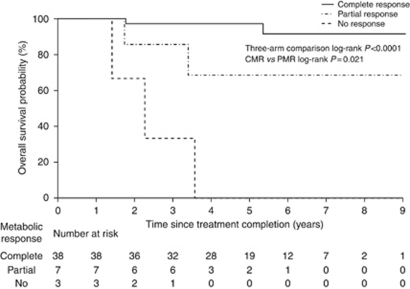
Cause-specific survival in all patients according to FDG-PET metabolic response.

Overall survival curves according to metabolic response (Figure 2) show differences in OS by metabolic response (*P*<0.0001 for three-arm comparison, *P*=0.03 for CMR *vs* PMR). The estimated OS rate at 5 years was 88% (95% CI: 78–100%) in patients with a CMR, 69% (95% CI: 40–100%) with PMR and 0% (95% CI: 0–71%) for no metabolic response. In the patient subgroup with PET scans within 40–140 days, OS results were again significant for the three-arm comparison (*P*<0.0001) but no longer reached significance for CMR *vs* PMR (*P*=0.09; data not shown). Anal CSS results stratified for metabolic response (Figure 3) were very similar to those for OS; *P*<0.0001 for three-arm comparison and *P*=0.02 for CMR *vs* PMR.

### Cox regression analyses

For PFS, the only significant correlation was with incomplete metabolic response to treatment (partial or no response) *vs* no response (HR 4.1. (95% CI: 1.5–11.5, *P*=0.013; Table 3). Higher T and N stage were suggestive of worse prognosis but did not reach statistical significance in this patient cohort. In prediction of OS times, FDG-PET incomplete metabolic response was again the only variable to show significant evidence of an association (HR 6.7 (95% CI: 2.1–21.6, *P*=0.002)).

## Discussion

The purpose of this study was to determine the utility of post-treatment FDG-PET in predicting outcomes in anal cancer managed with definitive chemoradiotherapy. To our knowledge, only one other publication has examined the value of FDG-PET in this setting, and found in a retrospective series of 53 patients (4 non-squamous histology) that metabolic response was a more significant predictor of PFS than pretreatment tumour size and nodal status (Schwarz *et al*, 2008). At a mean follow-up of 26 months, a significant difference in cause-specific survival rates was already evident between patients with a CMR *vs* PMR (2-year cause-specific survivals of 95% and 22%, respectively, *P*=0.0008) (Schwarz *et al*, 2008). Nguyen *et al* (2008) examined 25 patients who underwent pre- and post-chemoradiotherapy FDG-PET scans in a cohort of 50 patients retrospectively assessed for the impact of PET on their staging and management, but reported only descriptive findings; 2-year PFS was 68% in patients with CMR *vs* 40% in those with PMR.

A highly significant difference in PFS according to CMR, PMR or NR to chemoradiotherapy was seen in this series. If validated in other series, it could be postulated that a potential application of post-chemoradiotherapy FDG-PET is in identifying those patients with only a PMR for additional treatment, such as surgical intervention or enrolment in a clinical trial of novel therapies. Such an application of FDG-PET is the subject of current clinical trials in Hodgkin's and non-Hodgkin's lymphomas (Moskowitz *et al*, 2010) because of the highly prognostic nature of metabolic response during, and after, first-line treatment in these diseases (Mikhaeel *et al*, 2000; Kobe *et al*, 2008). Conversely, post-therapy FDG-PET may be of use in excluding the need for further investigation, such as biopsy, in patients with uncertain clinical findings, such as residual masses that may represent either viable tumour or radiotherapy-induced fibrosis, but a CMR. FDG-PET has proven valuable post chemoradiotherapy for SCC of the head and neck in identifying those patients who do not require a subsequent neck dissection despite residual lymphadenopathy (Porceddu *et al*, 2005; Nayak *et al*, 2007; Ong *et al*, 2008). In this setting, the negative predictive value of FDG-PET for residual malignancy at the primary site, within persistent cervical lymphadenopathy, and within the clinically node-negative neck is 97%, 94% and 98%, respectively (Ong *et al*, 2008).

Cause-specific and OS analyses found significant differences between all three categories of metabolic response and also specifically CMR *vs* PMR. NR patients (*n*=3) experienced early distant disease progression and poor survival times. The two patients in our cohort with baseline metastatic disease, although low volume and nonvisceral, were among the three nonresponders. As a result, the two-arm comparison of CMR *vs* PMR OS and cause-specific survival is an analysis of patients with locoregional disease only; the significant separation in the survivals of these two groups excludes the NR group as solely accounting for the highly significant *P-*value (*P*<0.0001) in the three-arm comparative results.

Of the 13 deaths in this study, 6 (46%) were not related to anal cancer, and yet did not preclude the demonstration of significantly different OS according to tumour metabolic response. Patients with only PMR or NR to chemoradiotherapy did not receive further curative-intent treatment on the basis of FDG-PET result alone because of the investigational nature of this imaging modality at the time and the presence of incurable distant disease in some patients. As such, this patient cohort provides observational data that may become limited in the future. Based on our experience in other diseases, salvage therapies are increasingly being instituted in patients with only PMR after definitive chemoradiation.

Primary tumour size and involvement of nodal basins are accepted anal cancer prognostic factors (Deniaud-Alexandre *et al*, 2003; Bilimoria *et al*, 2009; Myerson *et al*, 2009). These variables were recently validated prospectively in the RTOG 98–11 clinical trial cohort (Ajani *et al*, 2010). A recently published study in 77 patients with anal cancer found that pretreatment FDG-PET maximum standardised uptake value (SUVmax) may also confer prognostic information; higher baseline SUVmax showed borderline statistical association with disease-free survival (*P*=0.05); however, it was not associated with cause-specific survival (Kidd *et al*, 2010). In our study examining FDG-PET metabolic response, similar to Schwarz *et al* (2008), a greater correlation was seen with survival outcomes than for tumour T and N stages. These findings suggest that tumour metabolic response provides a valuable additional tool in prognostication. Previous studies in anal cancer have demonstrated that the clinical response within the primary anal tumour provides prognostic information (Chapet *et al*, 2005), and our results are consistent with this. The advantages of post-therapy FDG-PET over clinical examination, however, are the ability to simultaneously compare pre- and post-treatment assessments, ease of differentiating between abnormalities and normal tissue, and additional information provided regarding regional and distant disease status by a whole-body PET study.

The limitations of our study include its single-institution basis and retrospective nature, with resultant variability in the performance, and timing, of post-treatment imaging studies. The discrepancy in T stage between patients who underwent post-chemoradiotherapy FDG-PET scanning, compared with the whole cohort with a baseline FDG-PET study, did not create a significant stage difference between the patient groups, but may nonetheless limit the applicability of our findings to small, node-negative anal cancers. However, it could be argued that it is in patients with more advanced disease that therapeutic response assessment is most pertinent because of their higher risk of relapse. The optimal timing of post-therapy FDG-PET in anal cancer is currently unknown. In SCC of the head and neck, the negative predictive value of post-therapy metabolic response is higher than the positive predictive value because of confounding post-treatment inflammatory change (Porceddu *et al*, 2005; Nayak *et al*, 2007). In these two studies, as well as in another study (Andrade *et al*, 2006), later FDG-PET scans gave the greatest specificity. Because of the wide range of times to post-therapy FDG-PET scan in our study, a sensitivity analysis was conducted comparing scans within 40–140 days (71% patients) with all scans, and found identical proportions of CMR, PMR and NR in the respective groups. These findings would suggest that post-chemoradiotherapy FGD-PET findings in anal cancer are likely robust across a window period of some months; however, this issue clearly requires further investigation.

There remains scope for improvement in outcomes in both primary and salvage treatment for anal cancer. Definitive chemoradiotherapy with mitomycin and 5-FU is associated with a 5-year disease-free survival rate of 60% (Ajani *et al*, 2008). The current standard of care for patients with persistent or locally recurrent anal carcinoma is salvage APR, which yields 5-year OS rates, limited to patients managed with curative intent, of only 33–64% (Ellerhorn *et al*, 1994; Nilsson *et al*, 2002; Akbari *et al*, 2004; Mullen *et al*, 2006). The antiepidermal growth factor receptor (EGFR) monoclonal antibody cetuximab is in phase I clinical trial testing combined with cisplatin and 5-FU-based chemoradiotherapy (Olivatto *et al*, 2010). The incorporation of pre- and post-therapy FDG-PET in clinical trials of new treatment approaches in this malignancy would allow the prospective evaluation of the therapies, the significance of tumour metabolic response and appropriate timing of FDG-PET imaging.

In conclusion, this study has demonstrated the prognostic power of FDG-PET imaging in predicting survival outcomes in anal carcinoma managed with chemoradiotherapy. Given the findings of this and other studies, consideration should be given to incorporating measurement of FDG-PET metabolic response to treatment in prospective clinical trials in anal cancer.

## Figures and Tables

**Table 1:** Patient characteristics

**Baseline characteristic**	**All patients with baseline FDG-PET, *n* (%)**	**Patients with baseline and post-therapy FDG-PET, *n* (%)**	***P-*value**
No. of patients	74	48	
			
*Age (years)*			
Median	57	56	
Range	27–88	35–87	
			
*Gender*			
Female	44 (59)	26 (54)	0.23
Male	30 (41)	22 (46)	
			
*T stage*			
T1	24 (32)	9 (19)	0.02
T2	30 (41)	24 (50)	
T3	9 (12)	6 (13)	
T4	11 (15)	9 (19)	
			
*N stage*			
N0	47 (64)	30 (63)	0.53
N1	3 (4)	2 (4)	
N2	15 (20)	8 (17)	
N3	9 (12)	8 (17)	
			
*M stage*			
M0	72	46	
M1	2	2	
			
*Stage group*			
I	21 (28)	8 (17)	0.21
II	22 (30)	18 (38)	
IIIA	7 (9)	6 (13)	
IIIB	22 (30)	14 (29)	
IV	2 (3)	2 (4)	

Abbreviation: FDG-PET=^18^F-fluorodeoxyglucose positron emission tomography.

**Table 2:** Post-chemoradiotherapy metabolic responses grouped by timing of FDG-PET

**Result**	**All patients with post-treatment PET, *n* (%)**	**Patients with post-treatment PET in range 40–140 days, *n* (%)**
No. of patients	48	34
		
*Time to FDG-PET (days)*		
Median	69	73
Range	20–255	40–140
		
*Primary anal tumour*		
Complete response	41 (85)	29 (85)
Partial response	7 (15)	5 (15)
No response	0 (0)	0 (0)
		
*Regional lymph nodes*		
Complete response	48 (100)	34 (100)
Partial response	0 (0)	0 (0)
No response	0 (0)	0 (0)
		
*Distant disease*		
Complete response	45 (94)	32 (94)
Partial response	0 (0)	0 (0)
No response	3 (6)	2 (6)
		
*Overall tumour response rate*		
Complete response	38 (79)	27 (79)
Partial response	7 (15)	5 (15)
No response	3 (6)	2 (6)

Abbreviation: FDG-PET=^18^F-fluorodeoxyglucose positron emission tomography.

**Table 3:** Cox proportional hazards regression model for factors influencing progression-free and overall survival

**Variable**	**Hazard ratio (95% confidence interval (CI))**	***χ* ^2^**	***P-*value**
*Progression-free survival*			
Gender (male *vs* female)	0.7 (0.3–1.8)	0.6	0.43
Age	1.0 (1.0–1.0)	0.0	0.93
T stage	1.5 (0.9–2.4)	2.7	0.10
N stage	1.4 (0.9–2.0)	2.6	0.11
Incomplete metabolic response	4.1 (1.5–11.5)	6.2	0.013
			
*Overall survival*			
Gender (male *vs* female)	0.6 (0.2–1.8)	1.0	0.32
Age	1.0 (1.0–1.1)	0.4	0.52
T stage	1.6 (0.9–2.7)	3.0	0.09
N stage	1.5 (1.0–2.3)	9.0	0.08
Incomplete metabolic response	6.7 (2.1–21.6)	9.2	0.002

Incomplete metabolic response partial metabolic response (PMR) +no response (NR) *vs* complete metabolic response.

## References

[bib1] Ajani JA, Winter KA, Gunderson LL, Pederson J, Benson AB, Thomas CR, Mayer RJ, Haddock MG, Rich TZ, Willett C (2008) Fluorouracil, mitomycin and radiotherapy vs fluorouracil, cisplatin and radiotherapy for carcinoma of the anal canal. JAMA 299: 1914–1921 18430910 10.1001/jama.299.16.1914

[bib2] Ajani JA, Winter KA, Gunderson LL, Pederson J, Benson AB, Thomas CR, Mayer RJ, Haddock MG, Rich TA, Willett CG (2010) Prognostic factors derived from a prospective database dictate clinical biology of anal cancer: the Intergroup Trial (RTOG 98–11). Cancer 116: 4007–4013 20564111 10.1002/cncr.25188 PMC3831519

[bib3] Akbari RP, Paty PB, Guillem JG, Weiser MR, Temple LK, Minsky BD, Saltz L, Wong WD (2004) Oncologic outcomes of salvage surgery for epidermoid carcinoma of the anus initially managed with combined modality therapy. Dis Colon Rectum 47: 1136–1144 15164245 10.1007/s10350-004-0548-5

[bib4] Andrade RS, Heron DE, Degirmenci B, Filho PAA, Branstetter BF, Seethala RR, Ferris RL, Avril N (2006) Posttreatment assessment of response using FDG-PET/CT for patients treated with definitive radiation therapy for head and neck cancers. Int J Radiat Oncol Biol Phys 65: 1315–1322 16750327 10.1016/j.ijrobp.2006.03.015

[bib5] Benard F, Smith R, Hustinx P, Karp K, Alavi A. (1999) Clinical evaluation of processing techniques for attenuation correction with 137-Cs in whole-body PET imaging. J Nucl Med 40: 1257–1263 10450675

[bib6] Bilimoria KY, Bentrem DJ, Rock CE, Stewart AK, Ko CY, Halverson A (2009) Outcomes and prognostic factors for squamous-cell carcinoma of the anal canal: analysis of patients from the National Cancer Data Base. Dis Colon Rectum 52: 624–631 19404066 10.1007/DCR.0b013e31819eb7f0

[bib7] Borzomati D, Valeri S, Ripetti V, Vincenzi B, Rabitti C, Persichetti P, Valentini V, Trodella L, Caricato M, Coppola R (2005) Persisting perianal ulcer after radiotherapy for anal cancer: recurrence of disease or late radiation-related complication? Hepatogastroenterology 52: 780–784 15966204

[bib8] Chapet O, Gerard J-P, Riche B, Alessio A, Mornex F, Romestaing P (2005) Prognostic value of tumor regression evaluated after first course of radiotherapy for anal canal cancer. Int J Radiat Oncol Biol Phys 63: 1316–1324 16169674 10.1016/j.ijrobp.2005.05.047

[bib9] Cotter SE, Grigsby PW, Siegel BA, Dehdashti F, Malyapa RS, Fleshman JW, Birnbaum EH, Wang X, Abbey E, Tan B, Kodner IJ, Hunt SR, Lowney JK, Mutch MG, Dietz DW, Myerson RJ (2006) FDG-PET/CT in the evaluation of anal carcinoma. Int J Radiat Oncol Biol Phys 65: 720–725 16626889 10.1016/j.ijrobp.2006.01.009

[bib10] de Winton E, Heriot AG, Ng M, Hicks RJ, Hogg A, Milner A, Leong T, Fay M, MacKay J, Drummond E, Ngan SY (2009) The impact of 18-fluorodeoxyglucose positron emission tomography on the staging, management and outcome of anal cancer. Br J Cancer 100: 693–700 19259091 10.1038/sj.bjc.6604897 PMC2653751

[bib11] Deniaud-Alexandre E, Touboul E, Tiret E, Sezeur A, Houry S, Gallot D, Parc R, Huang R, Qu S-H, Huart J, Pene F, Schlienger M (2003) Results of definitive irradiation in a series of 305 epidermoid carcinomas of the anal canal. Int J Radiat Onc Biol Phys 56: 1259–1273 10.1016/s0360-3016(03)00417-6 12873670

[bib12] Duong C, Hicks R, Weih L, Drummond E, Leong T, Michael M, Thomas R (2006) FDG-PET status following chemoradiotherapy provides high management impact and powerful prognostic stratification in esophageal cancer. Eur J Nucl Med Mol Imaging 33: 770–778 16550384 10.1007/s00259-005-0040-z

[bib13] Ellerhorn DI, Enker WE, Quan SH (1994) Salvage abdominoperineal resection following combined chemotherapy and radiotherapy for epidermoid carcinoma of the anus. Ann Surg Oncol 1: 105–110 7834434 10.1007/BF02303552

[bib14] Frisch M, Glimelius B, van den Brule AJ, Wohlfahrt J, Meijer CJ, Walboomers JM, Goldman S, Svensson C, Adami HO, Melbye M (1997) Sexually transmitted infection as a cause of anal cancer. N Engl J Med 337: 1350–1358 9358129 10.1056/NEJM199711063371904

[bib15] Hicks RJ (2009) Role of ^18^F-FDG PET in assessment of response in non-small cell lung cancer. J Nucl Med 50(Suppl): 31S–42S 19380411 10.2967/jnumed.108.057216

[bib16] Holly EA, Whittemore AS, Aston DA, Ahn DK, Nickoloff BJ, Kristiansen JJ (1989) Anal cancer incidence: genital warts, anal fissure or fistula, hemorrhoids, and smoking. J Natl Cancer Inst 81: 1726–1731 2810388 10.1093/jnci/81.22.1726

[bib17] Iagaru A, Kundu R, Jadvar H, Nagle D (2009) Evaluation by 18F-FDG-PET of patients with anal squamous cell carcinoma. Hell J Nucl Med 12: 26–29 19330178

[bib18] Johnson LG, Madeleine MM, Newcomer LM, Schwartz SM, Daling JR (2004) Anal cancer incidence and survival: the surveillance, epidemiology, and end results experience, 1973–2000. Cancer 101: 281–288 15241824 10.1002/cncr.20364

[bib19] Juweid ME, Stroobants S, Hoekstra OS, Mottaghy FM, Dietlein M, Guermazi A, Wiseman GA, Kostakoglu L, Scheidhauer K, Buck A, Naumann R, Spaepen K, Hicks RJ, Weber WA, Reske SN, Schwaiger M, Schwartz LH, Zijlstra JM, Siegel BA, Cheson BD (2007) Use of positron emission tomography for response assessment of lymphoma: consensus of the imaging subcommittee of international harmonization project in lymphoma. J Clin Oncol 25: 571–578 17242397 10.1200/JCO.2006.08.2305

[bib20] Kalff V, Ware R, Heriot A, Chao M, Drummond E, Hicks RJ (2009) Radiation changes do not interfere with postchemoradiation restaging of patients with rectal cancer by FDG PET/CT before curative surgical therapy. Int J Radiat Oncol Biol Phys 74: 60–66 18922649 10.1016/j.ijrobp.2008.06.1944

[bib21] Kidd EA, Dehdashti F, Siegel BA, Grigsby PW (2010) Anal cancer maximum F-18 fluorodeoxyglucose uptake on positron emission tomography is correlated with prognosis. Radiother Oncol 95: 288–291 20231040 10.1016/j.radonc.2010.02.019

[bib22] Kobe C, Dietlein M, Franklin J, Markova J, Lohri A, Amthauer H, Klutmann S, Knapp WH, Zijlstra JM, Bockisch A, Weckesser M, Lorenz R, Schreckenberger M, Bares R, Eich HT, Mueller RP, Fuchs M, Borchmann P, Schicha H, Diehl V, Engert A (2008) Positron emission tomography has a high negative predictive value for progression or early relapse for patients with residual disease after first-line chemotherapy in advanced-stage Hodgkin lymphoma. Blood 112: 3989–3994 18757777 10.1182/blood-2008-06-155820 PMC2581984

[bib23] MacManus MP, Hicks RJ, Matthews JP, McKenzie A, Rischin D, Salminen EK, Ball DL (2003) Positron emission tomography is superior to computed tomography scanning for response-assessment after radical radiotherapy or chemoradiotherapy in patients with non-small cell lung cancer. J Clin Oncol 21: 1285–1292 12663716 10.1200/JCO.2003.07.054

[bib24] Mikhaeel NG, Timothy AR, O’Dpherty MJ, Hain S, Maisey MN (2000) 18-FDG-PET as a prognostic indicator in the treatment of aggressive non-Hodgkin's lymphoma: comparison with CT. Leuk Lymphoma 39: 543–553 11342337 10.3109/10428190009113384

[bib25] Moskowitz CH, Zelenetz A, Schoder H (2010) An update on the role of interim restaging FDG-PET in patients with diffuse large B-cell lymphoma and Hodgkin lymphoma. J Natl Compr Canc Netw 8: 347–352 20202464 10.6004/jnccn.2010.0023

[bib26] Mullen JT, Rodriguez-Bigas MA, Chang GJ, Barcenas CH, Crane CH, Skibber JM, Feig BW (2006) Results of surgical salvage after failed chemoradiation therapy for epidermoid carcinoma of the anal canal. Ann Surg Oncol 14: 478–483 17103253 10.1245/s10434-006-9221-7

[bib27] Myerson RJ, Outlaw ED, Chang A, Birnbaum EH, Fleshman JW, Grigsby PW, Kodner IJ, Malayapa RS, Mutch MG, Parikh P, Picus J, Tan BR (2009) Radiotherapy for epidermoid carcinoma of the anus: thirty years’ experience. Int J Radiat Onc Biol Phys 75: 428–435 10.1016/j.ijrobp.2008.11.047 19251377

[bib28] Nayak JV, Walvekar RR, Andrade RS, Daamen N, Lai SY, Argiris A, Ferris RL, Johnson JT, Branstetter BF (2007) Deferring planned neck dissection following chemoradiation for stage IV head and neck cancer: the utility of PET-CT. Laryngoscope 117: 2129–2134 17921898 10.1097/MLG.0b013e318149e6bc

[bib29] NCCN Clinical Practice Guidelines in Oncology™-V.1.2010 (www.nccn.org)

[bib30] Nguyen BT, Joon DL, Khoo V, Quong G, Chao M, Wada M, Joon ML, See A, Feigen M, Rykers K, Kai C, Zupan E, Scott A (2008) Assessing the impact of FDG-PET in the management of anal cancer. Radiother Oncol 87: 376–382 18453023 10.1016/j.radonc.2008.04.003

[bib31] Nigro M, Vaitkevicius V, Considine S (1974) Combined therapy for cancer of the anal canal: a preliminary report. Dis Colon Rectum 17: 354–356 4830803 10.1007/BF02586980

[bib32] Nilsson PJ, Svensson C, Goldman S, Glimelius B (2002) Salvage abdominoperineal resection in anal epidermoid cancer. Br J Surg 89: 1425–1429 12390386 10.1046/j.1365-2168.2002.02231.x

[bib33] Olivatto LO, Araujo CM, Vilhena B, Meton F, Bezerra M, Erlich F, Castro L, Ferreira CG (2010) Phase I study of cetuximab (CET) in combination with 5-FU, cisplatin (CP), and radiotherapy (RT) in patients with locally advanced squamous cell anal canal carcinoma (LASACC). 2010 Gastrointestinal Cancers Symposium. ASCO GI Congress 2010. Abstract No. 492

[bib34] Ong SC, Schoder H, Lee NY, Patel SG, Carlson D, Fury M, Pfister DG, Shah JP, Larson SM, Kraus DH (2008) Clinical utility of 18F-FDG PET/CT in assessing the neck after concurrent chemoradiotherapy for locoregional advanced head and neck cancer. J Nucl Med 49: 532–540 18344440 10.2967/jnumed.107.044792

[bib35] Palefsky JM, Holly EA, Ralston ML, Jay N, Berry JM, Darragh TM (1998) High incidence of anal high-grade squamous intra-epithelial lesions among HIV-positive and HIV-negative homosexual and bisexual men. AIDS 12: 495–503 9543448 10.1097/00002030-199805000-00011

[bib36] Porceddu SV, Jarmolowski E, Hicks RJ, Ware R, Weih R, Rischin D, Corry J, Peters LJ (2005) Utility of positron emission tomography for the detection of disease in residual neck nodes after (chemo)radiotherapy in head and neck cancer. Head Neck 27: 175–181 15627258 10.1002/hed.20130

[bib37] Robinson D, Coupland V, Moller H (2009) An analysis of temporal and generational trends in the incidence of anal and other HPV-related cancers in Southeast England. Br J Cancer 100: 527–531 19156144 10.1038/sj.bjc.6604871 PMC2658550

[bib38] Sato H, Koh P-K, Bartolo DCC (2005) Management of anal canal cancer. Dis Colon Rectum 48: 1301–1315 15793642 10.1007/s10350-004-0934-z

[bib39] Scherrer A, Reboul F, Martin D, Dupuy JC, Menu Y (1990) CT of malignant anal canal tumors. Radiographics 10: 433–453 2188307 10.1148/radiographics.10.3.2188307

[bib40] Schwarz JK, Siegel BA, Dehdashti F, Myerson RJ, Fleshman JW, Grigsby PW (2008) Tumor response and survival predicted by post-therapy FDG-PET/CT in anal cancer. Int J Radiat Oncol Biol Phys 71: 180–186 17996387 10.1016/j.ijrobp.2007.09.005

[bib41] Trautmann TG, Zuger JH (2005) Positron emission tomography for pretreatment staging and posttreatment evaluation in cancer of the anal canal. Mol Imaging Biol 7: 309–313 16028002 10.1007/s11307-005-0003-6

